# Taurine potentiates artemisinin efficacy against malaria by modulating the immune response in *Plasmodium berghei*-infected mice

**DOI:** 10.1186/s13071-024-06585-y

**Published:** 2024-11-29

**Authors:** Xin Li, Ning Jiang, Qilong Li, Kexin Zheng, Yiwei Zhang, Xiaoyu Sang, Ying Feng, Ran Chen, Qijun Chen

**Affiliations:** 1 Key Laboratory of Livestock Infectious Diseases, Ministry of Education, and Key Laboratory of Ruminant Infectious Disease Prevention and Control (East), Ministry of Agriculture and Rural Affairs, College of Animal Science and Veterinary Medicine, Shenyang Agricultural University, 120 Dongling Road, Shenyang, 110866 China; 2 Research Unit for Pathogenic Mechanisms of Zoonotic Parasites, Chinese Academy of Medical Sciences, 120 Dongling Road, Shenyang, 110866 China

**Keywords:** Taurine, Artemisinin, Suppress inflammation, Malaria, *Plasmodium*

## Abstract

**Background:**

Artemisinin (ART) is a frontline drug for the treatment of malaria; however, the emergence of ART-resistant *Plasmodium* strains necessitates increasing ART sensitivity. Given that taurine (TAU) has been shown to have immunomodulatory activity, we investigated the effects of TAU as an adjunct therapy to ART in mice infected with *Plasmodium berghei*.

**Methods:**

Mice infected with *P. berghei* ANKA strain (*P. berghei* ANKA) were treated with TAU alone, ART alone or a combination of TAU and ART (TAU + ART), and their survival time and parasitaemia were recorded. The cytotoxic effects of TAU and ART were subsequently assessed. The expression levels of inflammasome-related genes and inflammatory factors in mice infected with *P. berghei* ANKA were analysed in relation to those in mice treated with TAU alone, ART alone or the TAU + ART combination. The therapeutic effects were further evaluated by histological analysis and measurement of the spleen index.

**Results:**

Compared with the control mice, *P. berghei* ANKA-infected mice treated with ART in combination with TAU presented significantly lower parasitaemia and prolonged survival. The combined treatment resulted in significant reductions in the expression levels of inflammasome-related genes in the spleen, including absent in melanoma 2 (*AIM2*), caspase-1, NOD-, LRR- and pyrin domain-containing protein 3 (*Nlrp3*), *Nlrp1b*, *Nlrp1b*, NLR family CARD domain containing 4 (*Nlrc4*), *Nlrp6*, nucleotide binding oligomerization domain containing 1 (*NOD1*) and *NOD2*, and decreases in the levels of inflammatory cytokines in the serum, including interleukin (IL)-12p70, tumour necrosis factor-alpha, monocyte chemoattractant protein-1, IL-10 and IL-6. Histopathological analysis confirmed that TAU + ART combination treatment reduced spleen pathology caused by *P. berghei* ANKA infection.

**Conclusions:**

The findings indicate that TAU potentiates ART efficacy by modulating the immune response in *P. berghei*-infected mice.

**Graphical Abstract:**

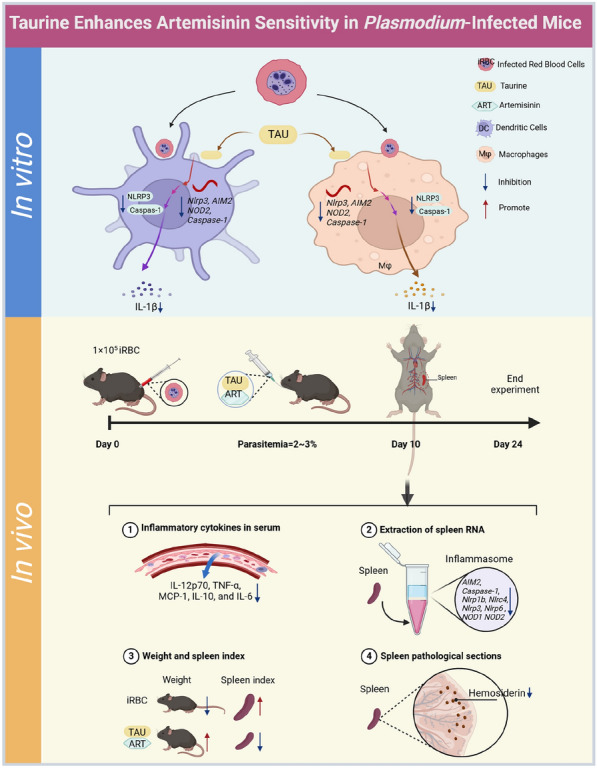

**Supplementary Information:**

The online version contains supplementary material available at 10.1186/s13071-024-06585-y.

## Background

Malaria significantly threatens the health of those living in malaria endemic areas [1]. The disease course is accompanied by a cytokine storm, leading to high fever, anaemia and organ failure [2]. An excessive production of cytokines, such as tumour necrosis factor-alpha (TNF-α), interferon-gamma (IFN-γ), interleukin (IL)-1 beta and IL-12 is associated with disease symptoms [3, 4]. IL-1β-mediated fever is a hallmark of malaria and is regulated by inflammasomes. Inflammasomes, which are multiprotein complexes, are activated during parasite infection, particularly NOD-, LRR- and pyrin domain-containing protein 3 (NLRP3) and absent in melanoma 2 (AIM2), which subsequently activate caspsase-1 and are responsible for IL-1β processing and secretion [5].

Artemisinin (ART) and its derivatives are among the most effective antimalarial drugs in terms of clearing various *Plasmodium* species [6–9]. Taurine (TAU), a semi-essential amino acid not incorporated into proteins, is abundant in mammalian tissues and innate immune cells such as lymphocytes [10], neutrophils [11], macrophages [12] and leukocytes [13]. It is also present in foods such as shellfish, red meat, organ meat, eggs, poultry and energy drinks [14]. TAU can be synthesized in humans from methionine via the homocysteine pathway [15]. Studies suggest that TAU, an anti-inflammatory molecule, helps balance macrophage polarization and reduce inflammatory damage [16, 17]. The protective effects of TAU have also been observed in rodent models of lung injury, central nervous system injury and chemical toxicity [18]. TAU treatment also reduces the expression levels of markers such as inducible nitric oxide synthase (iNOS), nuclear factor kappa-B (NF-κB), TNF-α and IL-2 during acute inflammation and has been shown to reduce TNF-α, IL-1β and IL-6 levels in *Klebsiella* infections [19–22]. In parasitic infections, TAU can initiate protective inflammatory responses [23]. Thus, we postulate that TAU supplementation may help alleviate cytokine storms caused by *Plasmodium*, although its adjunctive effect on malaria treatment with ART remains unclear.

In this study, we first examined the impact of combining TAU and ART on parasitaemia and survival in *Plasmodium berghei*-infected mice. Next, we examined the impact of TAU on macrophages and dendritic cells (DCs) and analysed the changes in inflammatory cytokine levels after TAU treatment. Additionally, the spleen index, weight changes and histopathology were assessed to evaluate the protective effects of the TAU and ART combination therapy (TAU + ART) in *P. berghei*-infected mice.

## Methods

### Parasites

Wild-type* P. berghei* ANKA strain (*P. berghei* ANKA) was maintained in our laboratory for use in this study.

### Drug preparation

For the in vivo experiments, 0.5 g of carboxymethylcellulose (CMC) (Solarbio, Beijing, China; catalogue no. 9004-32-4) was dissolved in 100 ml of distilled water. A 10 ml aliquot of the CMC solution was used to dissolve 0.1 g of ART (Aladdin Scientific, Riverside, CA, USA; catalogue no. A110206, approximately 98% purity) or 0.1 g of TAU (Aladdin Biochemical Technology Co., Shanghai, China; catalogue no. T103829, approximately 99% purity). CMC was used as a solvent control. For the in vitro experiments, 28.2 mg of ART or 12.5 mg of TAU was dissolved in 10 ml of ethanol (Tianjin Zhongtian Chemical Co., Ltd., Tianjin, China; CAS no. 64-17-5) and diluted 100-fold with phosphate-buffered saline (PBS) to prepare a 100 μM ART or 100 μM TAU solution. A total of 10 μl of ethanol solution was added to 990 μl of PBS to make a 1% ethanol solution as a solvent control.

### Animals

Female C57BL/6 mice (WT; 6–8 weeks old) were obtained from Liaoning Chang Sheng Biological Technology Company (Benxi, China) and housed in a pathogen-free environment with free access to food and water. Fifty mice were randomly divided into five groups (*n* = 10* per* group) for the survival experiment. Forty additional mice were randomly divided into five groups (*n* = 8 *per* group) for cytokine and other analyses. For *Plasmodium* infection, 1 × 10^5^ *P. berghei* ANKA-infected red blood cells (iRBCs) suspended in 200 µl of PBS from donor mice were intraperitoneally injected into the experimental mice. Four of the five groups were orally administered 1 mg ART, 1 mg TAU, 1 mg ART + TAU or 0.5 mg CMC daily when parasitaemia reached 2–3%. The dosages of both ART and TAU were determined based on previous studies [24, 25]. On the 10th day after parasite infection, tail tip blood was collected from each mouse, and the serum from each mouse was isolated. Daily parasitaemia and mortality rates were recorded until the mice were sacrificed at 24 days post infection.

### Cell lines and cultivation

RAW264.7 cells (Haixing Biotechnology Co., Ltd., Suzhou, China; catalogue no. 6210 TCM-C766) and DCs (Qingqi Biotechnology Development Co., Ltd., Shanghai, China; catalogue no. BFN680341) were cultivated in RPMI-1640 medium (HyClone, Waltham, MA, USA; catalogue no. SH30255.01) supplemented with 10% foetal bovine serum (FBS; Gibco, Thermo Fisher Scientific, Waltham, MA, USA; catalogue no. 10099141C) and 0.5% penicillin‒streptomycin solution (100×) (Beyotime Biotech Inc., Haimen, Jiangsu, China; catalogue no. C0222) at 37 °C in a humidified 5% CO_2_ atmosphere. These cell lines were regularly tested for mycoplasma contamination.

### Cell cytotoxicity assay

The cytotoxicity of ART and TAU to DCs and RAW264.7 (a mouse macrophagic cell line) cells was assessed using Cell Counting Kit-8 (CCK-8; Beyotime Biotech Inc.). The cells were seeded into 96-well plates at a density of 1 × 10^3^ cells per well and incubated for 24 h. ART or TAU, dissolved in fresh medium, was added to each well, followed by incubation for 24 or 48 h. The samples were then washed with PBS, and 10 µl of CCK-8 reagent was added to each well, following which the samples were incubated for 2 h. The absorbance was measured at 450 nm using a microplate reader (model 680; Bio-Rad Laboratories, Hercules, CA, USA). Cell viability was calculated as: cell inhibition rate (%) = 1 − [(A-C)/(B-C)] × 100, where A is the absorbance of the treated cells, B is the absorbance of the untreated cells and C is the absorbance of the blank medium.

### Quantitative real-time PCR

Dendritic cells and RAW264.7 cells were plated at 2 × 10^5^ cells per well in 6-well plates and grown overnight, followed by treatment with 25 µM ART, 25 µM TAU or 1% ethanol for 24 h. Total RNA samples were extracted from the cells using TRIzol reagent (Invitrogen, Thermo Fisher Scientific; catalogue no. 15596018) and converted to complementary DNA (cDNA) with the Takara First-Strand cDNA Synthesis Kit (Takara, Tokyo, Japan; catalogue no. 6210A). Real-time quantification of the messenger RNA (mRNA) templates was performed using Takara TB Green Premix Ex Taq™ (Takara; catalogue no. RR420A). The transcript-specific primers used in this study are listed in Additional file 1: Table S1. The comparative Ct method was used to quantify the target gene expression normalized to that of β-actin and relative to the calibrator; the data are expressed as the fold change (FC) = 2^−ΔΔCt.^

### Western blot analysis

Western blotting was performed on DCs and RAW264.7 cells plated at 2 × 10^5^ cells per well in 6-well plates and grown overnight. The cells were then incubated with iRBCs (cells = 1:10) for 12 h before the addition of 25 µM ART, 25 µM TAU, 25 µM ART + TAU or 1% ethanol, followed by 24 h of cultivation. Total proteins from the cells were extracted using lysis buffer (Shenggong, Shanghai, China), separated by 10% sodium dodecyl sulphate‒polyacrylamide gel electrophoresis (SDS-PAGE) and then transferred onto PVDF membranes (Bio-Rad Laboratories). The membranes were blocked with 5% nonfat milk in PBST (PBS with Tween 20) for 1 h at 37 °C, incubated with primary antibodies overnight at 4 °C, washed and incubated with horseradish peroxidase-conjugated goat anti-rabbit immunoglobulin G secondary antibodies (Beyotime). The target proteins were quantified based on band density with ImageJ 1.46r software. The antibodies used for western blotting are listed in Additional file 1: Table S2.

### Analysis of cytokines in mouse serum by cytometric bead array)

Sera were collected from the mice treated with ART or TAU. Cytokines, including IL-6, IL-10, monocyte chemoattractant protein-1 (MCP-1), IL-12p70 and TNF-α, were measured with a BD CBA Mouse Inflammation Kit (BD Biosciences, San Jose, CA, USA). Briefly, 50 µl of each serum sample was incubated with a mixture of mouse cytokine capture bead suspension and phycoerythrin (PE) detection reagent at 25 °C for 3 h. The samples were then washed twice with PBS, centrifuged at 300 *g* for 5 min at 4 °C and resuspended in 300 µl of PBS. Analysis was conducted using a fluorescence-activated cell sorting Aria III flow cytometer and BD CBA software (both BD Biosciences). Recombinant cytokine standards were diluted and used in parallel with the samples to prepare standard curves. The detection limits were: IL-6 > 5 pg/ml; IL-10 > 17.5 pg/ml; MCP-1 > 52.7 pg/ml; IL-12p70 > 10.7 pg/ml; and TNF-α > 7.3 pg/ml.

### Histological analysis

For the histological analysis, the spleens of drug-treated mice were surgically removed and fixed in 10% formalin. The spleen samples were then embedded in paraffin, sectioned into 3-µm-thick slices and mounted on glass slides. The sections were stained with haematoxylin and eosin (H&E) and examined under a microscope for histopathological changes. The haemosiderin content in the spleen tissue was determined using ImageJ 1.46r software.

### Statistical analysis

Statistical analyses were conducted using GraphPad Prism 7 software (GraphPad Software Inc., La Jolla, CA, USA). The results were analysed using one-way analysis of variance for multiple group comparisons, followed by Tukey's multiple comparisons test. For nonparametric data, the Kruskal‒Wallis Test was employed, followed by Dunn's multiple comparisons test. Survival curves were generated and compared using the log-rank (Mantel‒Cox) test. The area under the curve was calculated to evaluate parasitaemia between the experimental and control groups. Cytokine levels were analysed using FCAP Array Software (v3.0; BD Biosciences).

## Results

### TAU enhances the therapeutic effect of ART in *P. berghei* ANKA-infected mice

**Fig. 1:**
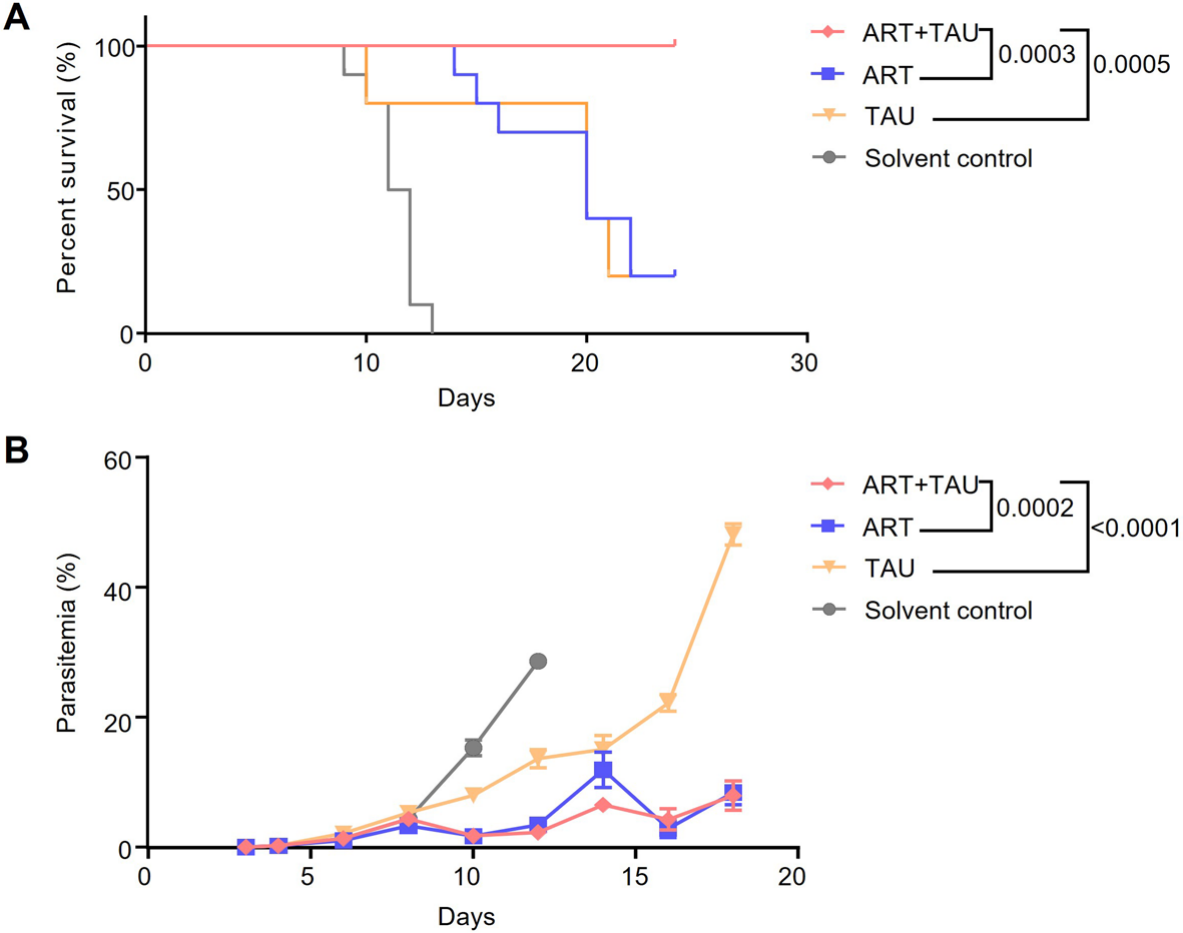
Combined treatment with TAU and ART effectively prolonged the survival time of mice infected with *Plasmodium berghei* ANKA strain and reduced parasitaemia. **A**, **B** Wild-type (WT) C57BL/6 mice were intraperitoneally infected with *P. berghei* ANKA strain (1 × 10^5^ infected red blood cells). The survival time of the mice in the ART + TAU group was significantly prolonged (**A**) and parasitaemia was significantly reduced (**B**) compared to that in the ART only and TAU only groups.* n* = 10 mice* per* group. The data represent the mean ± standard error of the mean.* P* values were calculated by the log-rank (Mantel‒Cox) test (**A**) and by the area under the curve (**B**). ART, Artemisinin; ART + TAU, ART and TAU co-therapy; TAU, taurine

To study the impact of TAU on ART treatment, we orally administered TAU and ART to *P. berghei* ANKA-infected mice and monitored survival time and parasitaemia. The survival time of the mice that were co-administered TAU + ART was significantly prolonged (Fig. 1a) while that in mice in the groups only treated with TAU or ART began to die 10 or 14 days post-infection. The survival time in the TAU and ART groups, respectively, was significantly shorter than that in mice receiving TAU + ART (Fig. 1a). Additionally, the parasitaemia in the TAU + ART group was significantly lower than that in the groups treated with only TAU or only ART (Fig. 1b). These results indicate that TAU can enhance the therapeutic effect of ART in mice infected with *P. berghei* ANKA.

### TAU reduced the expression of inflammatory factors in DCs and RAW264.7 cells

**Fig. 2:**
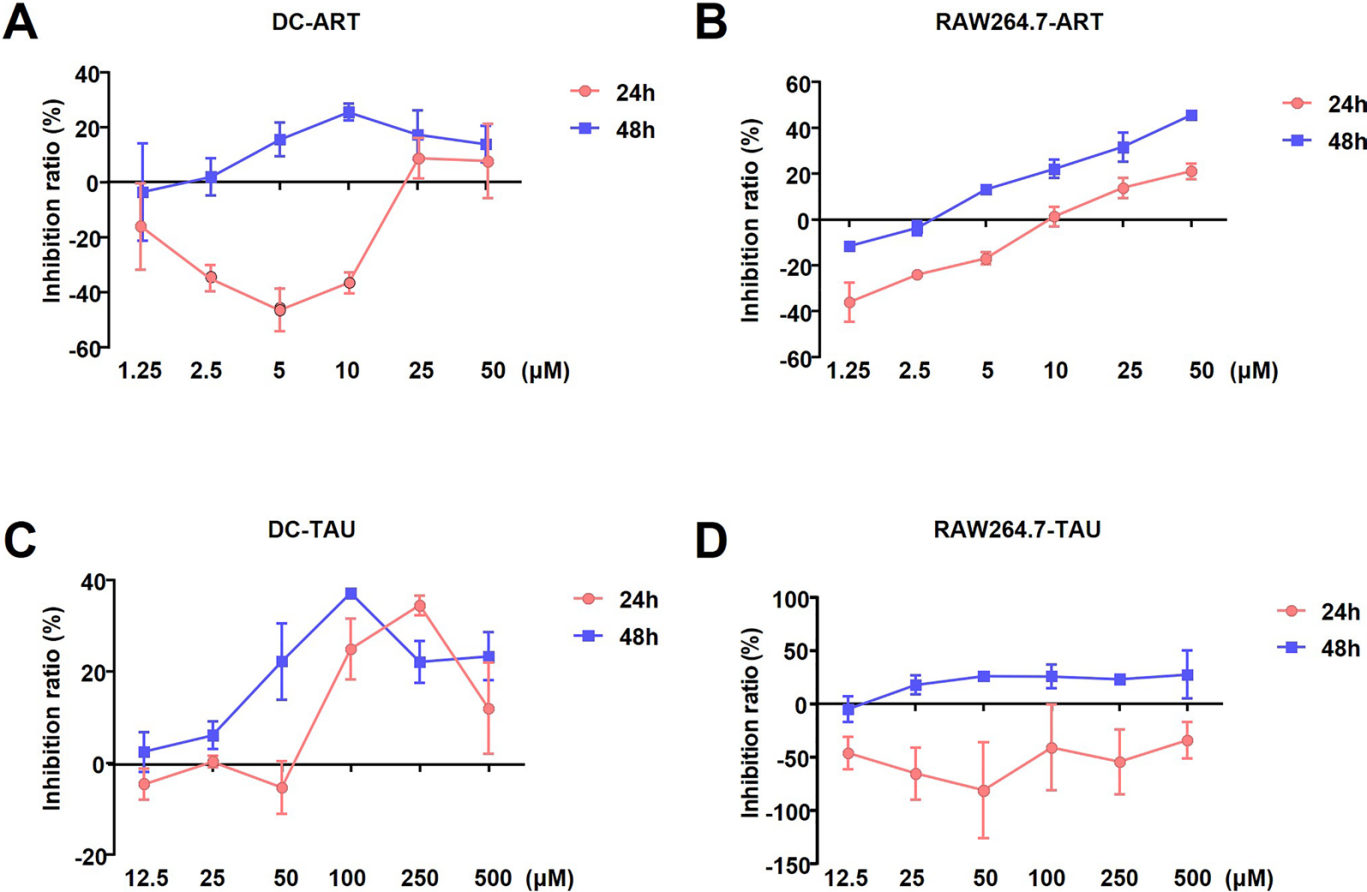
TAU did not significantly inhibit the growth of DCs or RAW264.7 cells. **A**,** B **The cytotoxicity of ART (0–50 μM) to DCs (**A**) and RAW264.7 mouse macrophages (**B**). **C**,** D** The cytotoxicity of TAU (0–500 μM) to DCs (**C**) and RAW264.7 cells (**D**). *n* = 3 biological replicates. The data represent the means ± standard error of the mean. ART, Artemisinin; ART + TAU, ART and TAU co-therapy; DCs, dendritic cells; TAU, taurine

To investigate the potential mechanisms by which TAU enhances the therapeutic effects of ART, we first examined the inhibitory effects of TAU and ART on innate immune cells, including DCs and RAW264.7 macrophages. Elevated concentrations of ART resulted in more pronounced inhibition at 48 h than at 24 h (Fig. 2a, b). Conversely, TAU exhibited differential effects, as evidenced by negative inhibition in RAW264.7 cells at 24 h, indicating a lack of cytotoxicity towards these cells (Fig. 2c, d). Consequently, a concentration of 25 μM TAU with a 24-h incubation period was selected for subsequent experiments.

**Fig. 3:**
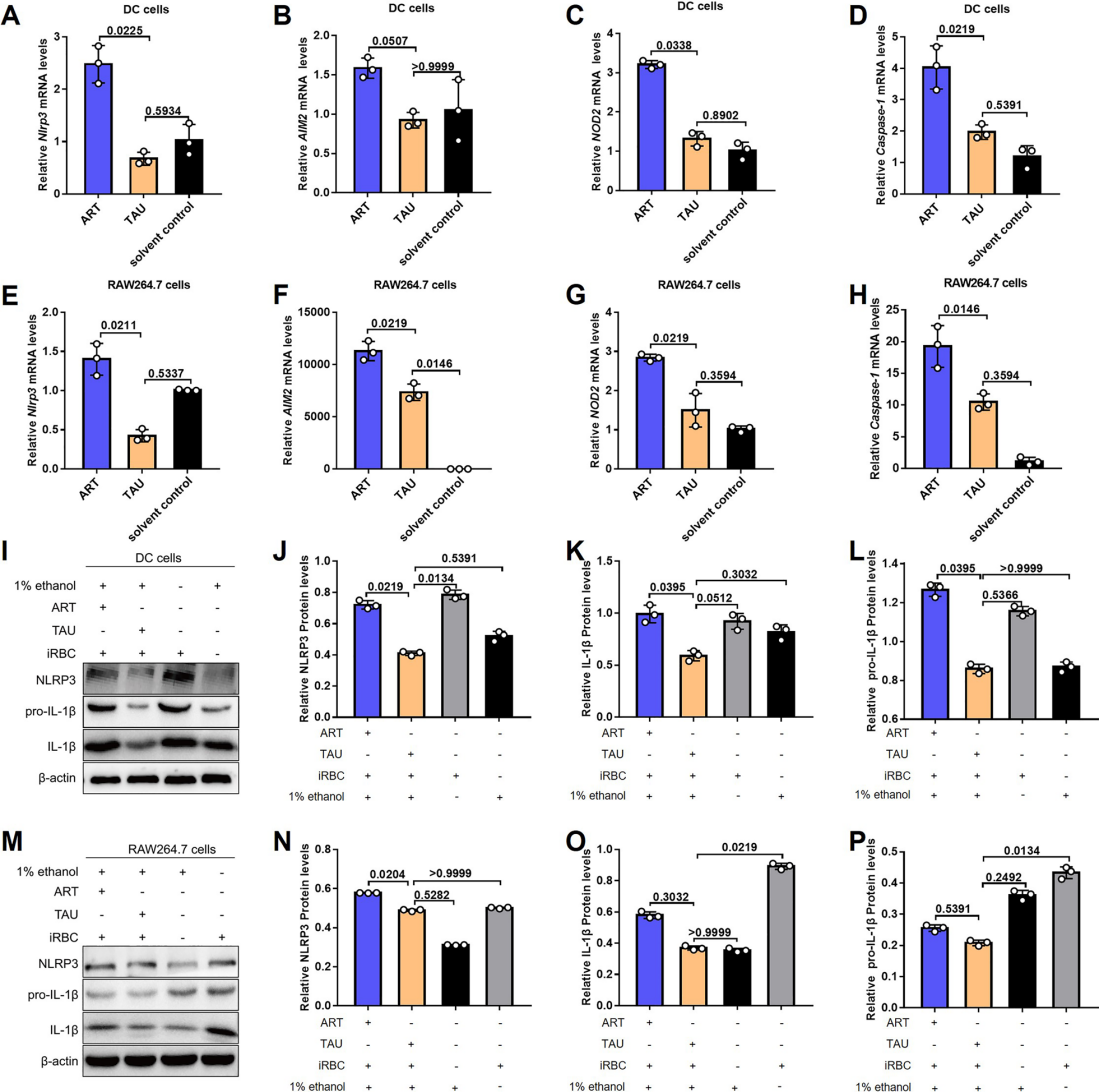
TAU reduced the expression of inflammatory factors in DCs and RAW264.7 cells. **A** Relative transcription levels of *Nlrp3* in DCs treated with 25 μM ART, TAU or the solvent control. **B** Relative transcription levels of *AIM2* in DCs treated with 25 μM ART, TAU or the solvent control. **C** Relative transcription levels of *NOD2* in DCs treated with 25 μM ART, TAU or the solvent control. **D** Relative transcription levels of *caspase-1* in DCs treated with 25 μM ART, TAU or the solvent control. **E** Relative transcription levels of *Nlrp3* in RAW264.7 cells treated with 25 μM ART, TAU, or the solvent control. **F** Relative transcription levels of *AIM2* in RAW264.7 cells treated with 25 μM ART, TAU or the solvent control. **G** Relative transcription levels of *NOD2* in RAW264.7 cells treated with 25 μM ART, TAU or the solvent control. **H** Relative transcription levels of *caspase-1* in RAW264.7 cells treated with 25 μM ART, TAU or the solvent control. **I** Expression levels of the proteins NLRP3, IL-1β and pro-IL-1β in DCs treated with 25 μM ART, TAU or 1% ethanol. **J** Relative expression levels of NLRP3 in DCs after 24 h of treatment with 25 μM ART, TAU or 1% ethanol. **K** Relative expression levels of IL-1β in DCs after 24 h of treatment with 25 μM ART, TAU or 1% ethanol. **L** Relative expression levels of pro-IL-1β in DCs after 24 h of treatment with 25 μM ART, TAU or 1% ethanol. **M** Expression levels of the NLRP3, IL-1β and pro-IL-1β proteins in RAW264.7 cells treated with 25 μM ART, TAU or 1% ethanol for 24 h. **N** Expression levels of NLRP3 in RAW264.7 cells treated with 25 μM ART, TAU, or 1% ethanol for 24 h. **O** Relative expression levels of IL-1β in RAW264.7 cells treated with 25 μM ART, TAU or 1% ethanol for 24 h. **P** Relative expression levels of pro-IL-1β in RAW264.7 cells treated with 25 μM ART, TAU or 1% ethanol for 24 h. *n* = 3 biological replicates. * P*-values, shown above bars, were calculated using the Kruskal‒Wallis test. The data represent the mean ± standard deviation of the mean.* AIM2*, Absent in melanoma 2 gene; ART, artemisinin; IL, interleukin; DCs, dendritic cells; iRBCs, *P. berghei* ANKA-infected red blood cells; mRNA, messenger RNA;* NOD2*, nucleotide binding oligomerization domain containing 2 gene,* Nlrp3*, NLRP3, NOD-, LRR- and pyrin domain-containing protein 3 gene, protein; TAU, taurine

Administration of TAU has the potential to modulate the expression levels of inflammasome-related genes, including *Nlrp3*,* AIM2*, nucleotide binding oligomerization domain containing (* NOD2*) and *caspase-1*, in DCs (Fig. 3a–d) and RAW264.7 cells (Fig. 3e–h). TAU treatment also significantly reduced the expression levels of NLRP3, IL-1β, pro-IL-1β, caspase-1 and pro-caspase-1 in both DCs and RAW264.7 cells after stimulation with iRBCs (Fig. 3i‒p; Additional file 1: Figure S1). These results suggest that TAU can reduce the expression levels of inflammatory factors in innate immune cells.

### Combined treatment with TAU and ART resulted in reduced expression of inflammatory cytokines in *P. berghei* ANKA-infected mice

**Fig. 4:**
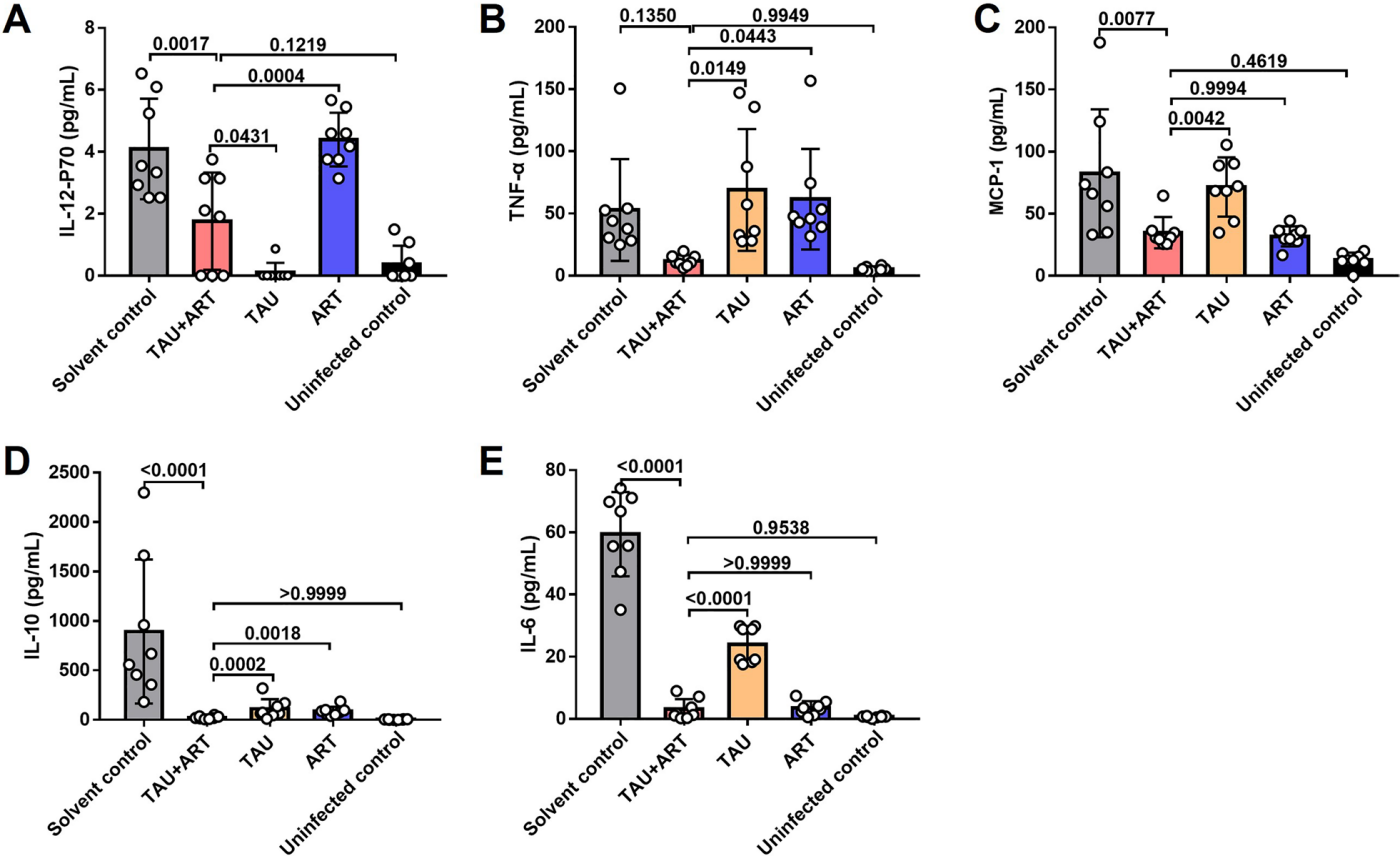
Treatment with TAU in combination with ART reduced the inflammatory cytokine levels in the sera from *Plasmodium berghei* ANKA-infected mice. **A**–**E** Serum levels of IL-12p70 (**A**), TNF-α (**B**), MCP-1 (**C**), IL-10 (**D**) and IL-6 (**E**). *n* = 8 mice.* P*-values, shown above bars, were calculated using one-way analysis of variance. The data represent the mean ± standard deviation of the mean. ART, Artemisinin; ART + TAU, ART and TAU co-therapy; IL, interleukin; MCP-1, monocyte chemoattractant protein-1; TAU, taurine; TNF-α, tumour necrosis factor alpha

**Fig. 5:**
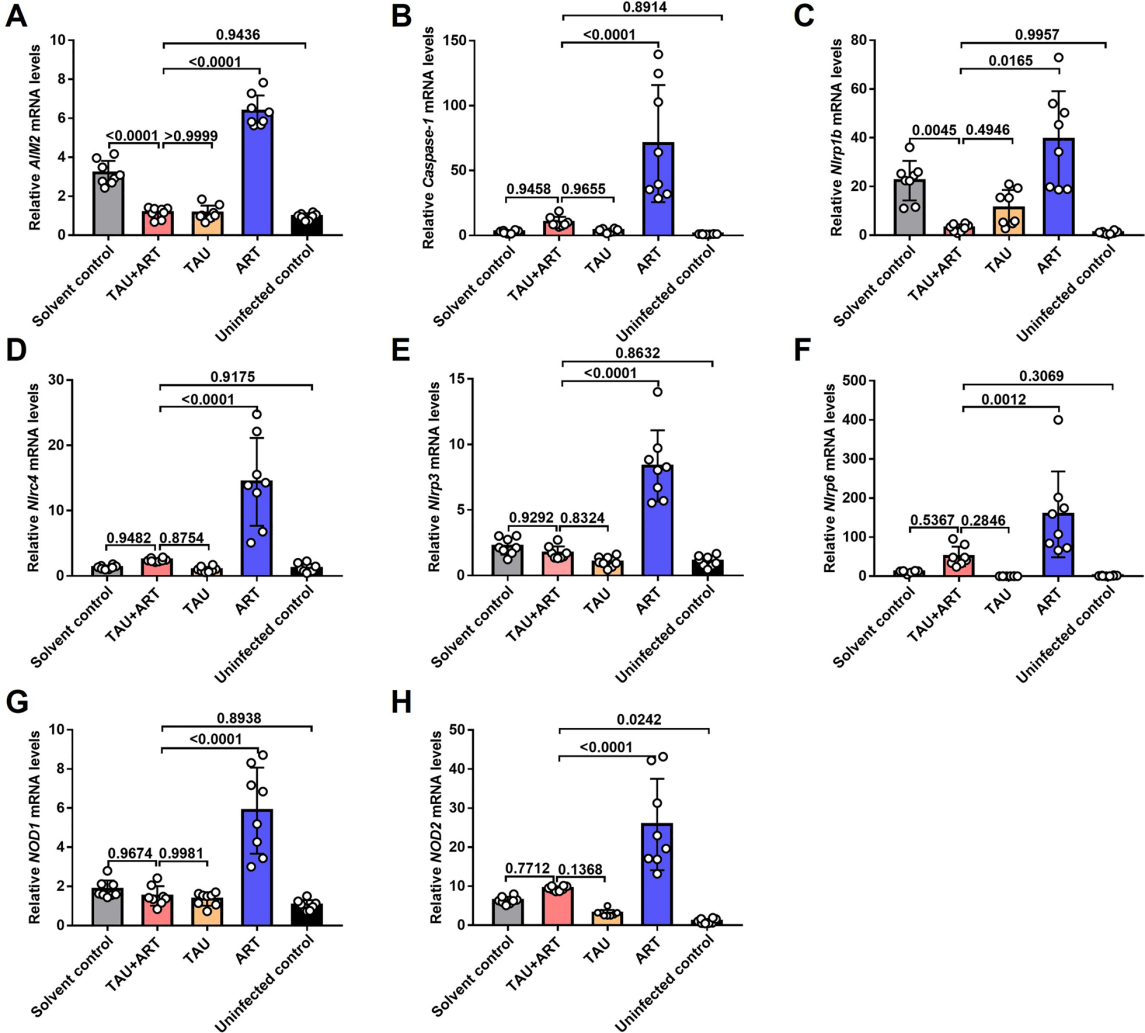
Treatment with TAU reduced the expression of inflammasome genes in ART-treated *P. berghei* ANKA-infected mice. **A**–**E** The relative transcription levels of the genes *AIM2* (**A**), *Caspase-1* (**B**), *Nlrp1b* (**C**), *Nlrc4* (**D**), *Nlrp3* (**E**), *Nlrp6* (**F**), *NOD1* (**G**) and *NOD2* (**H**) from the different groups are shown. *n* = 8 mice.* P*-values, shown above bars, were calculated using one-way analysis of variance. The data represent the mean ± standard error of the mean. * AIM2*, Absent in melanoma 2 gene; ART, Artemisinin; ART + TAU, ART and TAU co-therapy; * Nlrc4*, NLR family CARD domain containing 4 gene; * Nlrp3*, NOD-, LRR- and pyrin domain-containing protein 3 gene,* Nlrp6*, NOD-, LRR- and pyrin domain-containing protein 6; * NOD1, NOD2*, nucleotide binding oligomerization domain containing 1, 2 gene; TAU, taurine

To further investigate the role of TAU in mice infected with *P. berghei* ANKA, we examined the expression levels of inflammatory cytokines in the sera of mice treated with the TAU + ART combination. Compared with the solvent control treatment, combined TAU + ART treatment significantly reduced the serum levels of the cytokines IL-12p70, MCP-1, IL-10 and IL-6 in the mice. Notably, the expression levels of IL-12p70, TNF-α and IL-10 in the TAU + ART group were significantly lower than those in the ART**-**treated group (Fig. 4). Furthermore, compared with the expression levels in the ART**-**treated group, those of the genes *AIM2*, *Caspase-1*, *Nlrp1b*, NLR family CARD domain containing 4 (*Nlrc4*), *Nlrp3*, *Nlrp6*, *NOD1* and *NOD2* were significantly lower in the spleens of the mice in the TAU + ART group (Fig. 5). These results indicate that TAU reduces the expression of inflammatory cytokines.

### Combined treatment with TAU and ART alleviated the spleen pathology caused by *P. berghei* ANKA infection

**Fig. 6:**
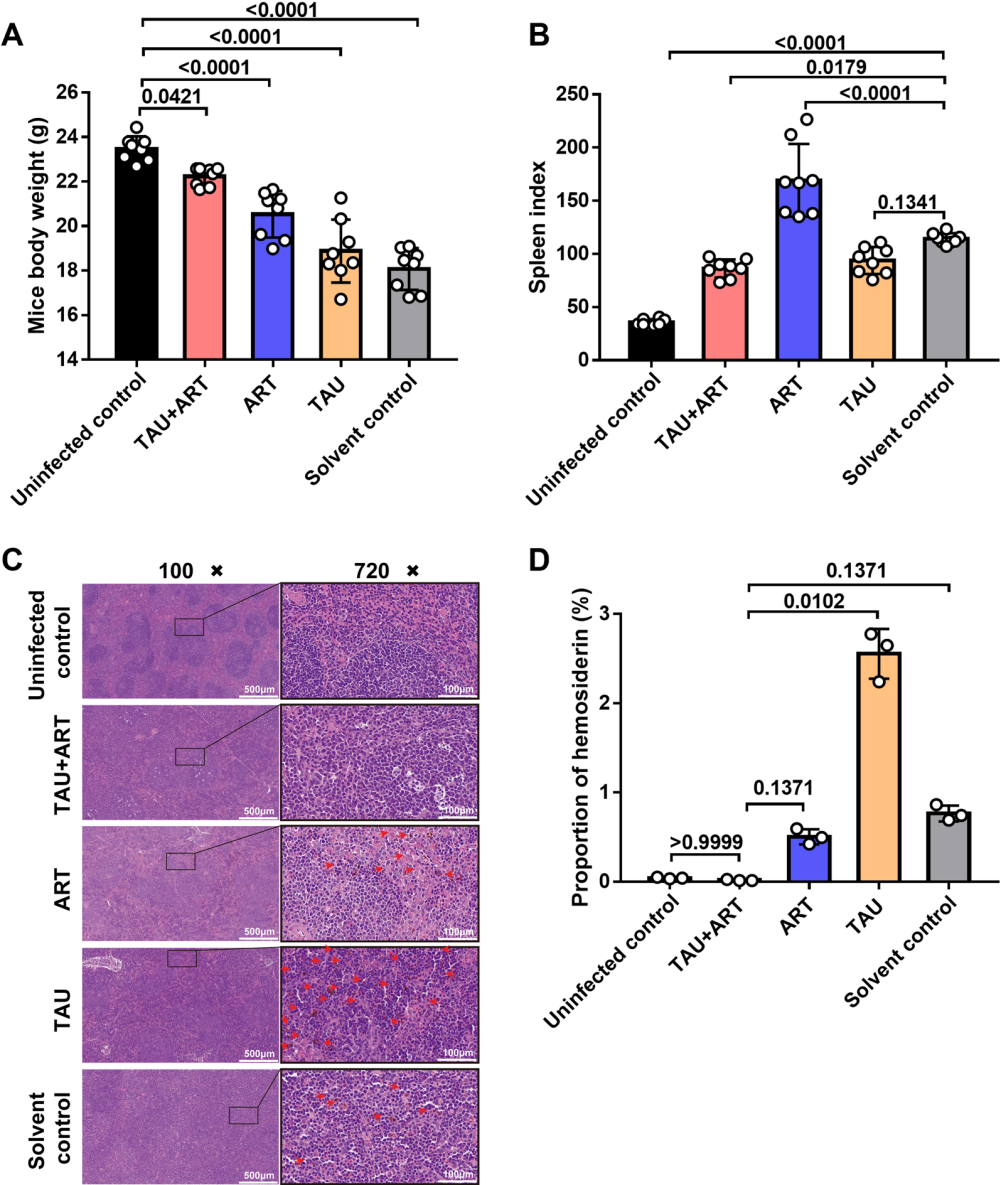
Treatment with TAU alleviated the spleen pathology resulting from *Plasmodium berghei* ANKA infection. **A** Body weights of the mice in the five groups (*n* = 8 mice per group). **B** Spleen indices of the five groups (*n* = 8 mice per group). *P*-values in** A** and** B**, shown above bars, were calculated using one-way analysis of variance. **C** Haematoxyln and eosin staining results for the spleens. **D** Histogram of the areas of spleen tissues containing haemosiderin (*n* = 3 biological replicates). The *P* values in** D**, shown above bars, were calculated using the Kruskal‒Wallis test. The data represent the mean ± standard error of the mean. ART, Artemisinin; ART + TAU, ART and TAU co-therapy; TAU, taurine

We conducted comprehensive analyses of the body weight, spleen index and spleen histopathology of the infected mice after TAU + ART treatment. The body weights of the mice treated with the combined TAU + ART therapy remained relatively stable compared with those of the uninfected control group, whereas the mice treated with either ART alone or TAU alone exhibited significant weight loss (Fig. 6a). Additionally, the spleen index of the TAU + ART-treated group was markedly lower than that of the solvent control group (Fig. 6b). Furthermore, the haemosiderin content in the spleen tissues of the TAU + ART group was significantly lower than that of the TAU group and was comparable to that of the uninfected control group (Fig. 6c, d). These findings suggest that TAU + ART combination therapy mitigates not only the expression of inflammatory cytokines in infected mice but also the pathology induced by the parasite.

## Discussion

Previous studies have shown that host immune responses during *Plasmodium* infection can be intense, resulting in a cytokine storm [26]. The driver of this storm is the *Plasmodium* infection, which can induce the secretion of various proinflammatory cytokines, such as IL-12 and IL-10 [27, 28]. A recent study indicated that supplementary therapy with TAU reduces the ratio of myeloid to lymphoid cells, significantly decreasing the serum levels of TNF-α, IL-17, IL-1β and granulocyte‒macrophage colony‒stimulating factor (GM-CSF) [29]. In the present study, we treated *Plasmodium*-infected mice with subtherapeutic doses of ART and found that supplementation with TAU significantly increased the survival time of the infected mice and reduced parasitaemia (Fig. 1). Further investigation revealed that TAU did not inhibit the growth of macrophages or DCs (Fig. 2) or reduce the expression of inflammatory factors induced by iRBC-stimulated macrophages or DCs (Fig. 3). Additionally, in mice treated with ART, TAU supplementation not only significantly reduced the levels of inflammatory cytokines in the peripheral blood but also decreased the expression of inflammasome genes in the spleen (Figs. 4, 5). Moreover, compared with the uninfected control group, the TAU + ART group did not exhibit significant weight loss, showed no significant differences in splenic haemosiderin levels and had a significantly lower spleen index than the solvent control group (Fig. 6). Overall, our results suggest that TAU protects the host by reducing inflammatory responses, thereby enhancing the therapeutic effect of ART against malaria.

Measuring the survival time and parasitaemia of mice are important criteria for evaluating the effectiveness of malaria treatment. In the present study, mice infected with *P. berghei* ANKA were given oral TAU + ART, and their survival time was significantly longer than those of mice given ART alone or TAU alone (Fig. 1a). The combined treatment with TAU and ART also significantly reduced parasitaemia (Fig. 1b).

Previous studies have indicated that TAU not only is the most abundant free amino acid in innate immune cells but also acts as an anti-inflammatory agent in the treatment of various diseases [30]. Innate immune cells, such as macrophages and DCs, perform various innate immune defence functions by recognizing pathogens and activating inflammasomes [31]. Therefore, in the present study, we used DCs and RAW264.7 cells as models to study the potential mechanisms involved. We also found that the expression levels of inflammasome genes in macrophages and DCs in the TAU**-**treated group were significantly lower than those in the ART-treated group (Fig. 3a–h), accompanied by reductions in the expression levels of inflammatory proteins induced by *Plasmodium*-stimulated innate immune cells (Fig. 3i–p; Additional file 1: Figure S1). In mice infected with *P. berghei* ANKA and treated with subtherapeutic doses of ART, TAU supplementation significantly reduced the serum levels of the inflammatory cytokines IL-12p70, TNF-α, MCP-1, IL-10 and IL-6 (Fig. 4) and significantly decreased the expression levels of the inflammasome genes *AIM2*, *Caspase-1*, *Nlrp1b*, *Nlrc4*, *Nlrp3*, *Nlrp6*, *NOD1* and *NOD2* in the spleen (Fig. 5), consistent with the in vitro findings. These findings led us to conclude that TAU has anti-inflammatory effects during malaria treatment.

Weight loss during disease progression is commonly observed in mice infected with *P. berghei* ANKA [32, 33]. The spleen, as a complex secondary lymphoid organ, plays a crucial role in controlling the blood stage of *Plasmodium* infection [34]. It is known that *Plasmodium* induces abnormal immune responses, leading to splenomegaly. In the present study, mice in the TAU + ART oral administration group did not exhibit significant weight loss compared with those in the uninfected control group at 10 days post infection with *P. berghei* ANKA (Fig. 6a). However, the spleen index was also significantly lower in the TAU + ART group than in the solvent control group (Fig. 6b). Furthermore, histopathological analysis of HE-stained spleen sections revealed that the haemosiderin content in the spleens of the TAU + ART group was significantly lower than those in the other *P. berghei* ANKA-infected groups (Fig. 6c, d). These findings suggest that TAU can mitigate the damage caused by *Plasmodium* infection during ART treatment. All analyses were performed on female C57BL/6 mice infected with *P. berghei* ANKA.

Recent studies have demonstrated that elevated levels of TAU metabolites in human blood correlate with reduced levels of the inflammatory marker C-reactive protein [29]. Furthermore, TAU, when administered within a reasonable concentration range, has no toxic effects on humans and can be safely administered [25]. However, it must be noted that our study only supports the use of TAU as adjuvant therapy to ART and other antimalarials; TAU alone does not have any antimalarial effect beyond its immunomodulatory activity. Another limitation is that the results were obtained in a mouse model with *P. berghei* ANKA infection, a relevant model for *P. falciparum* malaria. Therefore, human trials are essential to determine whether TAU supplementation can enhance the therapeutic efficacy of ART in the treatment of malaria.

## Conclusions

Our study demonstrated that TAU can significantly reduce the inflammation caused by *Plasmodium* infection, improving the therapeutic efficacy of ART. This reduction resulted in longer survival, lower parasitaemia, less weight loss and less spleen damage in treated mice. These findings highlight the anti-inflammatory benefits and potential adjunctive effects of TAU in malaria treatment. Supplementation with TAU or TAU combined with antimalarial drugs may augment malaria treatment efficacy.

## Supplementary Information

Additional file 1: Figure S1. TAU reduces the expression of inflammatory cytokines. Table S1. Primers were used in this study. Table S2. Key antibodies were used in the study.

## Data Availability

All data supporting the findings of this study are available within the paper.
