# Supplementary material for: Taurine potentiates artemisinin efficacy against malaria by modulating the immune response in Plasmodium berghei-infected mice
Source: Parasit Vectors. 2024 Nov 29;17:493. doi: 10.1186/s13071-024-06585-y (PMC11606117; doi:10.1186/s13071-024-06585-y)
Supplement: Supplementary file 1 — Additional file 1: Figure S1. TAU reduces the expression of inflammatory cytokines. Table S1. Primers were used in this study. Table S2. Key antibodies were used in the study. [file 13071_2024_6585_MOESM1_ESM.docx]

**Taurine Potentiates Artemisinin Efficacy Against Malaria by Modulating Immune Response in *Plasmodium berghei*-Infected Mice**

Xin Li^1,2†^, Ning Jiang^1,2†^, Qilong Li^1,2†^, Kexin Zheng^1,2^, Yiwei Zhang^1,2^, Xiaoyu Sang^1,2^, Ying Feng^1,2^, Ran Chen^1,2^, Qijun Chen^1,2*^

^1^Key Laboratory of Livestock Infectious Diseases, Ministry of Education, and Key Laboratory of Ruminant Infectious Disease Prevention and Control (East), Ministry of Agriculture and Rural Affairs, College of Animal Science and Veterinary Medicine, Shenyang Agricultural University, 120 Dongling Road, Shenyang 110866, China

^2^Research Unit for Pathogenic Mechanisms of Zoonotic Parasites, Chinese Academy of Medical Sciences, 120 Dongling Road, Shenyang 110866, China


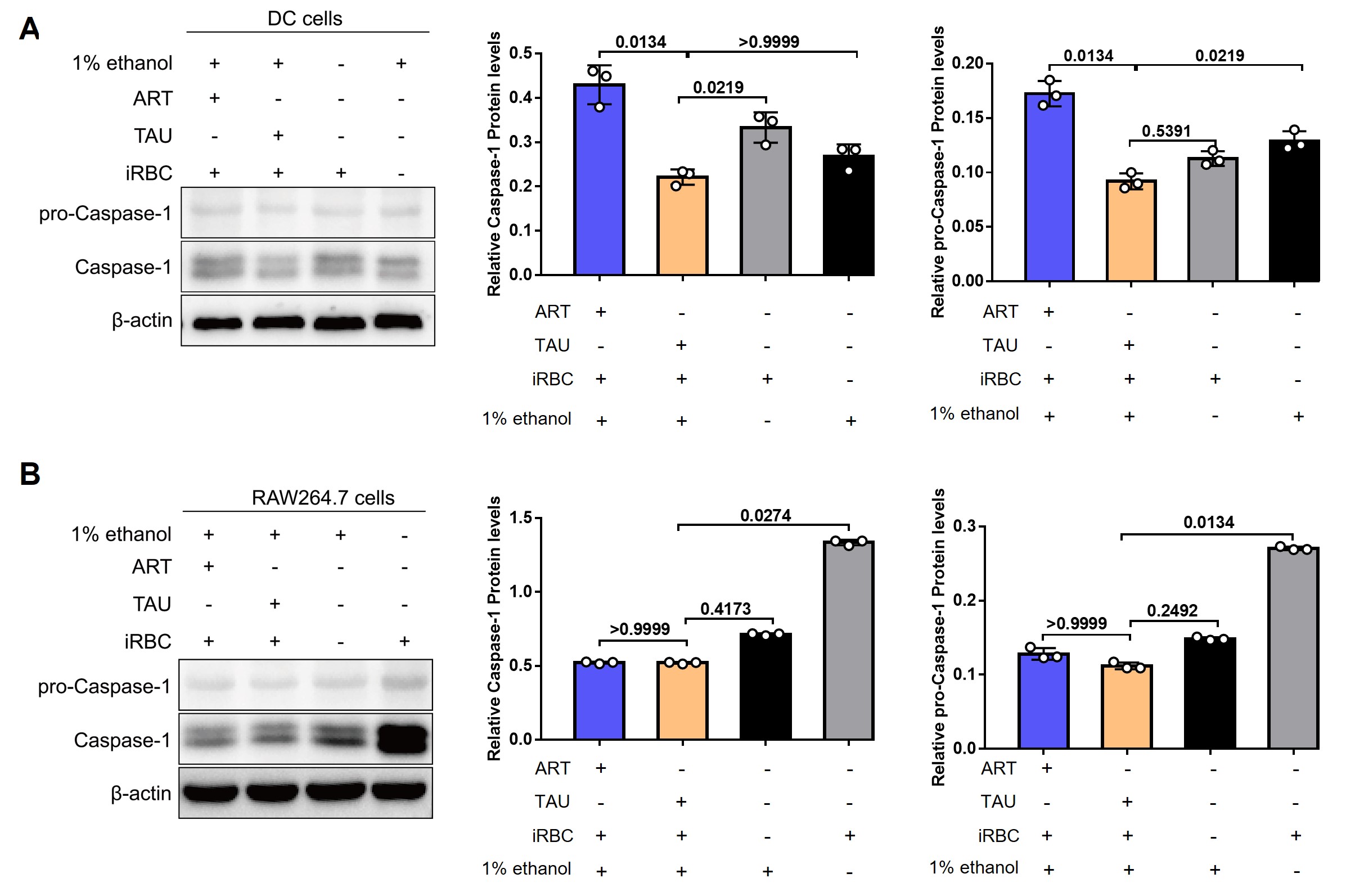


**Fig. S1.** **TAU reduces the expression of inflammatory cytokines (A)** Expression levels of the Caspase-1, and pro-Caspase-1 in DC cells treated with 25 uM ART, TAU or 1% ethanol for 24 h. **(B)** Expression levels of the Caspase-1, and pro-Caspase-1 in RAW264.7 cells treated with 25 uM ART, TAU or 1% ethanol for 24 h. *n*=3, biological replicates. **The *P*-value was calculated by Kruskal-Wallis Test. Data represent the mean ± s.e.m.**

**Table S1.** **Primers used in this study.**

| Primer name | Primer sequence (5’- 3’) |
| --- | --- |
| NLRP3-F | *ATTACCCGCCCGAGAAAGG* |
| NLRP3-R | *TCGCAGCAAAGATCCACACAG* |
| AIM2-F | *GTCACCAGTTCCTCAGTTGTG* |
| AIM2-R | *CACCTCCATTGTCCCTGTTTTAT* |
| NOD2-F | *CAGGTCTCCGAGAGGGTACTG* |
| NOD2-R | *GCTACGGATGAGCCAAATGAAG* |
| Caspase-1-F | *ACAAGGCACGGGACCTATG* |
| Caspase-1-R | *TCCCAGTCAGTCCTGGAAATG* |
| NLRP1b-F | *AGTAATCTGGAGGGGTTGGAC* |
| NLRP1b-R | *GTTGGCAGCCAGGGTATATCA* |
| NLRC4-F | *TTGAAGGCGAGTCTGGCAAAG* |
| NLRC4-R | *GGCGCTTCTCAGGTGGATG* |
| NLRP6-F | *CTCGCTTGCTAGTGACTACAC* |
| NLRP6-R | *AGTGCAAACAGCGTCTCGTT* |
| NOD1-F | *GAAGGCACCCCATTGGGTT* |
| NOD1-R | *AATCTCTGCATCTTCGGCTGA* |
| β-actin-F | *ACGGTCAGGTCATCACTATCG* |
| β-actin-R | *GGCATAGAGGTCTTTACGGATG* |

**Table S2.** **Key antibodies used in the study.**

| Reagent | Usage | Dilution ratio | Cat.# | Source |
| --- | --- | --- | --- | --- |
| IL-1β Polyclonal Antibody | Western Blot | 1:1000 | YT5201 | Immunoway |
| Caspase-1 Polyclonal Antibody | Western Blot | 1:1000 | YT5743 | Immunoway |
| NLRP3 Rabbit pAb | Western Blot | 1:1000 | A5652 | ABclonal |
| β-Actin Rabbit Monoclonal Antibody | Western Blot | 1:1000 | AF5003 | Beyotime |
